# Rate of individuals with clearly increased radiosensitivity rise with age both in healthy individuals and in cancer patients

**DOI:** 10.1186/s12877-018-0799-y

**Published:** 2018-05-04

**Authors:** Barbara Schuster, Anna Ellmann, Theresa Mayo, Judith Auer, Matthias Haas, Markus Hecht, Rainer Fietkau, Luitpold V. Distel

**Affiliations:** 1 Department of Radiation Oncology, University Hospital Erlangen Friedrich-Alexander-Universität Erlangen-Nürnberg, Universitätsstr. 27, 91054 Erlangen, Germany; 2 Department of Radiology, Charité Universitätsmedizin, Berlin, Germany

**Keywords:** Radiosensitivity, 3-colour fluorescence in situ hybridization, Chromosomal aberration, Breaks per metaphase, Individual radiosensitivity

## Abstract

**Background:**

The question of an age dependence of individual radiosensitivity has only marginally been studied so far. Therefore, we analyzed blood samples of healthy individuals and cancer patients of different ages to determine individual radiosensitivity.

**Methods:**

Ex vivo irradiated blood samples of 595 individuals were tested. Chromosomes 1, 2 and 4 were stained by 3-color fluorescence in situ hybridization and aberrations were analyzed. Radiosensitivity was determined by the mean breaks per metaphase (B/M).

**Results:**

Healthy individuals (mean age 50.7 years) had an average B/M value of 0.42 ± 0.104 and an increase of 0.0014B/M per year. The patients (mean age 60.4 years) had an average B/M value of 0.44 ± 0.150 and radiosensitivity did not change with age. In previous studies we found that from a value of 0.6B/M on an individual is considered to be distinctly radiosensitive. The portion of radiosensitive individuals (B/M > 0.6) increased in both cohorts with age.

**Conclusion:**

Individual radiosensitivity rises continuously with age, yet with strong interindividual variation. No age related increase of radiosensitivity can be demonstrated in patients due to the strong interindividual variation. However among old cancer patients there is a higher probability to have patients with clearly increased radiosensitivity than at younger age.

**Electronic supplementary material:**

The online version of this article (10.1186/s12877-018-0799-y) contains supplementary material, which is available to authorized users.

## Background

It is widely believed that human radiosensitivity increases with age due to an increase of oxidative stress, telomere attrition, a decline in DNA damage response efficiency and inflammatory response [1, 2]. Especially DNA double stand break repair seems to be affected. Studies with γH2Ax staining found that γH2Ax foci accumulated with older age reflecting unrepaired DNA damage [3–6]. However it is still unclear whether the remaining DNA double strand breaks result in an increased radiosensitivity along with a higher risk of radiotherapy related side effects. Clinical studies show contradictory results and are time-consuming [2, 7–10]. A possibility to estimate individual radiosensitivity is to study the occurrence of radiation induced chromosomal aberrations after ex vivo irradiation.

Since the end of the last century chromosomal aberrations were used to predict patient’s radiosensitivity with the aim to individualize dose or fractionation in clinical radiotherapy [11–14]. Several studies were able to predict individual radiosensitivity using different techniques [15–18]. Different time points were used to irradiate lymphocytes. In the G0 assay lymphocytes were irradiated ex vivo in the G0 phase and were stimulated afterwards [14] while in the G2 assay lymphocytes were stimulated first and irradiated afterwards [18]. Either metaphases were analyzed after staining all chromosomes by conventional stains [18] or some of the chromosomes were painted by fluorescence in situ hybridization [19]. Mostly two different scoring protocols were used to classify aberrations. The PAINT system by Tucker et al. [20] and the S&S system by Savage and Simpson [21]. In spite of all these differences in the majority of studies chromosomal aberrations were able to predict individual radiosensitivity [15–18, 22–25]. The reason why chromosomal aberration testing is not the standard assay of predicting radiosensitivity is probably the time consuming assay and the need of an expert to score the metaphases.

Lymphocytes of the peripheral blood are commonly used because they are easy to obtain. Furthermore, they are in the G0-phase and therefore no cell cycle dependent difference in radiosensitivity occurs. After ex vivo irradiation, lymphocytes have to pass through damage processing and control functions. These are at least repair of the different ionizing radiation induced DNA damages, signaling transduction, induction of cell death and cell cycle arrest. If these functions are impaired, this can lead to an increased radiosensitivity. After carrying out all these essential checks, lymphocytes can enter metaphase. Therefore chromosomal aberration analysis tests for the most important cellular functions and therefore this late endpoint has the potential to predict radiosensitivity based on different reasons.

Our approach was to study the radiosensitivity in dependence of age in a cohort of healthy individuals and a cancer patient cohort. In the healthy individual cohort we expected to have a lower proportion of outliers and get a clearer correlation of radiosensitivity and age. The cancer patient cohort is the relevant cohort for clinical radiotherapy. With both cohorts together we expected to clarify a possible age dependence of radiosensitivity.

## Methods

### Patients

Whole blood samples (heparinized blood) of 393 patients with various cancers and of 202 healthy individuals were drawn (Table 1). Healthy individuals were defined to have no history of a malignant disease and to have a Karnofsky performance status of at least 90. Cancer patients were defined to have or have had at least one malignant disease. For the study malignant neoplasms of the lymphatic and hematopoietic tissue (ICD-10, 200–208) and non-melanoma skin cancers (ICD-10, C44) were excluded. Rectal cancer (2009–2015), breast cancer (2011–2013) and head and neck cancer (2013–2014) patients were consecutively sampled in the university hospital of Erlangen-Nürnberg. All blood samples were taken prior to radiotherapy with the exception of twelve patients. For those post radiotherapy patients blood was taken at least 4 month after the end of radiotherapy. Parts of the breast cancer data (67 patients) and healthy individuals data (62) were previously published [26].

**Table 1:** Characteristics of patients and healthy individuals

	Patients	Healthy individuals
*n*	393	202
Gender (%)		
Male	208 (52.9)	90 (44.6)
Female	185 (47.1)	112 (55.4)
Age (years)		
Mean age	60.4	50.7
Range	17–91	9–81
Cancer patients (%)		
Rectal carcinoma	203 (51.6)	
Breast carcinoma	101 (25.7)	
Lung cancer	34 (8.7)	
Head and neck tumours	21 (5.3)	
Melanoma	8 (2)	
Prostate Cancer	3 (0.8)	
Other	23 (5.9)	

### Chromosome preparation

The patients’ blood was drawn prior to radiation therapy. Blood of each individual was divided into two portions. The control sample was sham irradiated, whereas the other portion was irradiated with a dose of 2 Gy by a 6-MV linear accelerator (Mevatron, Siemens, Germany). The lymphocytes were stimulated with phytohemagglutinin and cultivated in the incubator. Lymphocytes were grown for 48 h and were blocked in the metaphase by colcemid and chromosome preparations were performed. In order to detect chromosome 1, 2 and 4, the DNA was hybridized with chromosome specific probes. The staining was carried out by different fluorescent dyes and the whole DNA was counterstained with DAPI. The three color Fluorescence in situ hybridization (FiSH) was performed as previously described [14, 27, 28].

### Image acquisition and analysis

A fluorescence microscope (Zeiss, Axioplan 2, Göttingen, Germany) and the Metasystems software (Metafer 4 V3.10.1, Altlussheim, Germany) were used to search chromosome metaphase spreads automatically at 100× magnification and an image of each metaphase was acquired at a magnification of 630×. For each metaphase spread black and white images of each color (red, green and blue) were acquired and used for evaluation (Additional file 1: Figure S1). With support of an image analysis software (Biomas, Erlangen, Germany) at least 100 metaphases were analyzed and translocations, dicentric chromosomes, acentric chromosomes, rings, deletions, insertions and complex chromosomal rearrangements (CCRs) were scored and data transferred to a spreadsheet (Excel, Microsoft Corporation, Redmond, WA, USA) and scores (B/M) were calculated. The aberrations were scored by the number of underlying chromosomal breakages according to Savage and Simpson [21]. The B/M value of the irradiated sample was then corrected by the B/M value of the sham irradiated sample.

### Statistical analysis

The statistical analysis of the data was performed by SPSS Statistics 21 (IBM, Armonk, NY, USA) [29, 30]. Data were tested by the Levene-test and T-test, Pearson’s r correlation, Spearman’s rho correlation and Kolmogorov Smirnov test. Graphics were plotted using Excel (Microsoft Corporation, Redmond, WA, USA) or TechPlot 7 (SFTek, Braunschweig, Germany).

## Results

### Cohort of healthy individuals and cancer patients

In total, blood of 595 individuals of different ages was studied for radiosensitivity. The whole cohort consisted of 202 healthy individuals and 393 patients with various solid malignancies like rectal cancer, breast cancer, lung cancer, head and neck tumors, melanoma and prostate cancer (Table 1). The mean age of the healthy individuals was 50.7 years and the mean cancer patients’ age was 60.4 years.

### Three-color fluorescence in situ hybridization

**Fig. 1:**
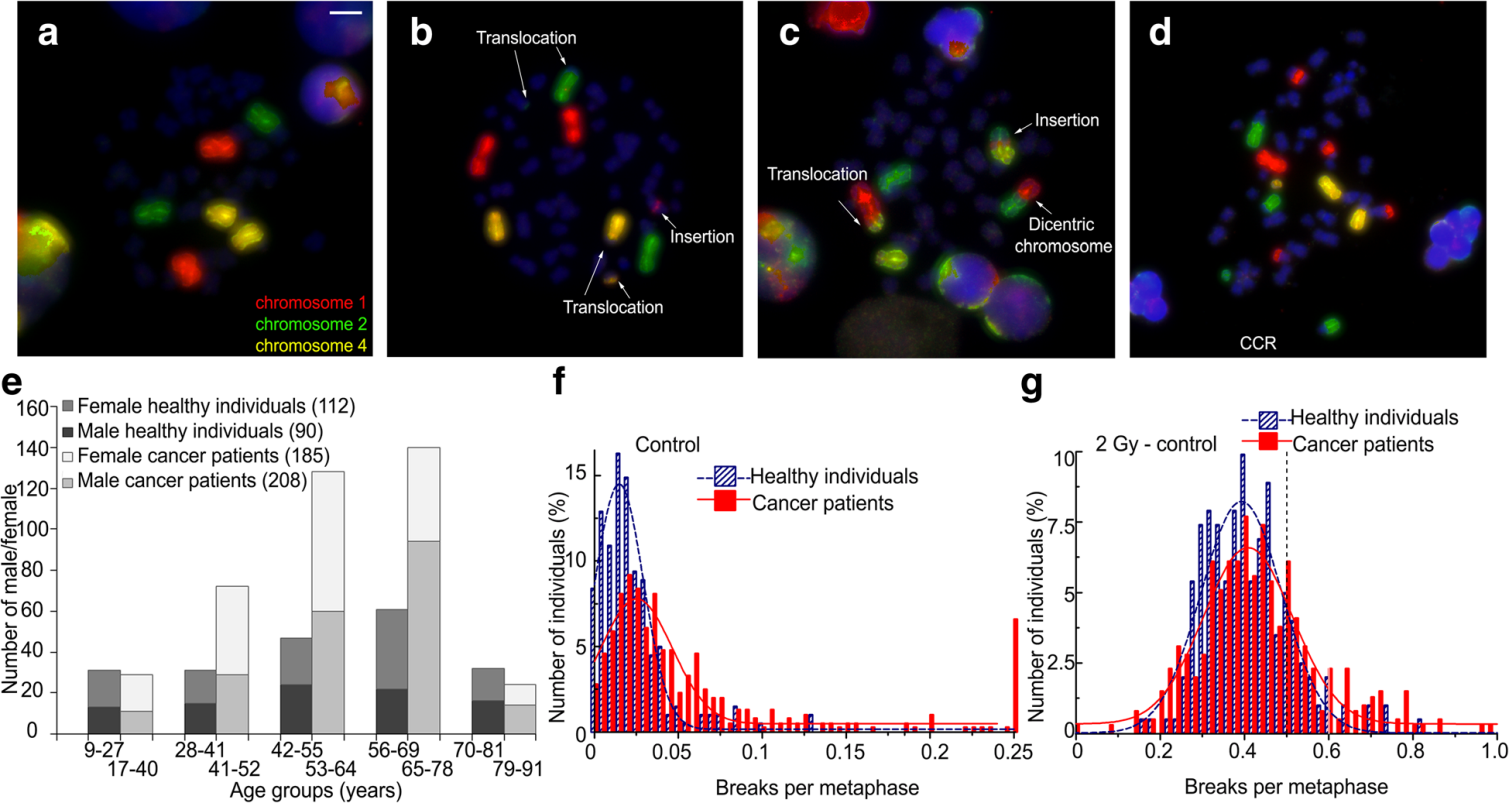
Three-color fluorescence in situ hybridization to determine radiosensitivity. Metaphase spreads of human blood lymphocytes, stained with chromosome specific probes for chromosome # 1 (red, rhodamine), chromosome # 2 (green, FITC) and chromosome # 4 (yellow, rhodamine + FITC). DNA was stained with DAPI (blue) (**a**-**d**). Normal metaphase spread (**a**). Metaphase spread with two translocations of # 2 with a blue chromosome and # 4 with a blue chromosome and an insertion of # 1 into a blue chromosome. The three aberrations were scored as 7 breaks (**b**). Metaphase spread with a dicentric chromosome of # 1 and # 2, a translocation of # 1 and # 4 and an insertion of # 1, # 2 and # 4. The aberrations were scored as 5 breaks (**c**). Metaphase spread with complex chromosomal rearrangements (CCR). The aberrations were scored as 12 breaks (**d**). Gender distribution of 202 healthy individuals in different age groups (9–27, 28–41, 42–55, 56–69 and 70–81 years) and of 393 patients with various cancer diseases (17–40, 41–52, 53–64, 65–78, 79–91 years) (**e**). Number of healthy individuals and cancer patients classified into divisions of 0.02 breaks per metaphase. Zero point five B/M (dashed line) and 0.6 B/M (solid line) are marked as a cutoff point to increased and distinctly increased radiosensitivity. B/M background without irradiation (**f**) and after irradiation of 2 Gy, corrected by the background (**g**). The data were fitted using a Gaussian distribution; scale 10 μm

Radiosensitivity was studied by a three color in situ hybridization assay. Peripheral blood was irradiated ex vivo by a dose of 2 Gy or left unirradiated. Lymphocytes were stimulated and arrested in the first cell division. Standard metaphases were prepared and the three-color FiSH assay was performed. Chromosomes 1, 2 and 4 of the metaphases were analyzed for chromosomal aberrations. Color changes along the three chromosomes were categorized as a chromosomal aberration and were scored according to as many break events were theoretically necessary for the constitution of the respective aberration. Acentric fragments, deletions and open breaks were scored as one break event; translocations, dicentrics and ring chromosomes were scored as two break events; insertions were scored as three breaks events and for complex chromosome aberrations the number of underlying break events was calculated (Fig. 1a-d) as proposed by Savage et al. [21]. The average aberration frequency is expressed as breaks per metaphase (B/M). In previous studies we found that from a value of 0.5 B/M on an increased radiosensitivity can be assumed and from a value of 0.6 B/M on an individual is considered to have a distinctly increased radiosensitivity [19, 28].

### B/M values and age in healthy individuals and cancer patients

We studied the distribution of B/M values in the healthy individuals and cancer patient cohort (Fig. 1f, g). A Gaussian fit for the healthy individuals was applied and the two times standard deviation was used as cut off. In the group of healthy individuals 10.4% and among cancer patients 25.7% were outliers, exceeding the two times standard deviation (Fig. 1f). Similarly, after 2 Gy ex vivo irradiation and correction by background levels we found more outliers in the patients’ group (11.5%) than in the group of healthy individuals (5.9%, Fig. 1g).

**Fig. 2:**
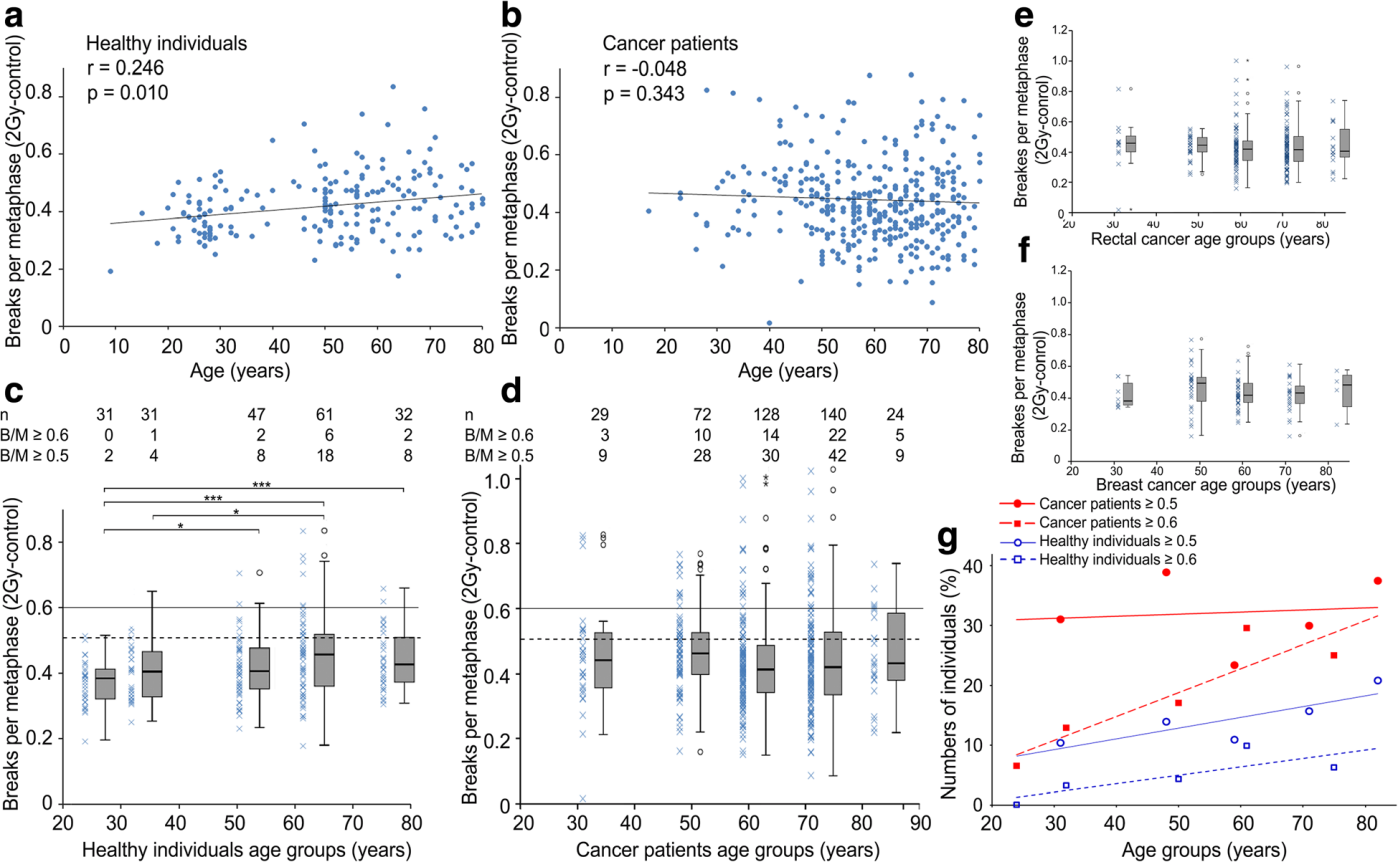
Radiosensitivity in healthy individuals and cancer patients of different age groups. Radiosensitivity, defined by chromosomal breaks per metaphase, in healthy individuals (**a**) and cancer patients (**b**) in dependence of age. A trend line was fitted and a Pearson correlation was performed (healthy individuals: *r* = 0.245, *p* < 0.01, cancer patients *r* = − 0.048, *p* < 0,343). 202 healthy individuals and 393 patients were divided into different groups by age: healthy individuals in groups with mean ages of 24, 32, 50, 61, 75 and patients in groups with mean ages of 32, 48, 59, 71, 82 years (**c**-**f**). 0.5 B/M (dashed line) and 0.6 B/M (solid line) are marked for increased and distinctly increased radiosensitivity. Healthy individuals (**c**), all cancer patients (**d**), the subgroup of rectal cancer patients (*n* = 203) (**e**) and breast cancer patients (*n* = 101) (**f**). Fraction of healthy individuals and cancer patients with equal or greater breaks per metaphase than 0.5 B/M or 0.6 B/M (**g**). *P*-values ≤0.05 are marked with one asterisk, P-values ≤0.01 are marked with two asterisks and P-values ≤0.001 are marked with three asterisks

Furthermore, the age of healthy individuals and the 2Gy B/M values (corrected) of each individual were tested for correlation. 2Gy B/M values increased clearly with age by 0.0014 B/M per year (*r* = 0.246, *p* = 0.01) in the healthy individual cohort. With a mean age of 50.7 years the healthy individual cohort had a mean B/M value of 0.42 ± 0.104. Mean B/M values raised from 0.39 at the age 30 to 0.44 B/M at the age 75 (Fig. 2a). In the patient cohort no correlation of age and B/M values was found (*p* = 0.343) and the slope of the line tended to be negative. The mean age of patients was 60.4 years with a mean B/M value of 0.44 ± 0.150 (Fig. 2b).

We presumed that there is an age dependency of B/M values and therefore divided the cohorts into different age groups. In average the cohort of healthy individuals was younger than the cancer patients’ group. To have sufficient numbers of individuals in each group we divided the cohorts into age groups of at least 24 individuals (Fig. 2c, d). The mean age of the healthy individuals groups was 24, 32, 50, 61 and 75 years, respectively, and of the cancer patients the mean age was 32, 48, 59, 71 and 82 years, respectively. B/M values were clearly increased when comparing the youngest healthy individuals group of 24 years to the older individuals of 50 (*p* = 0.027), 61 (*p* < 0.001) and 75 years (*p* = 0.001) (Fig. 2c). In the patient cohort B/M values do not differ between the age groups (Fig. 2d). In the cancer patient subgroups of rectal cancer patients (*n* = 203) (Fig. 2e) and breast cancer patients (*n* = 101) (Fig. 2f) was no change in radiosensitivity over age. Since more outliers were observed among cancer patients compared to the cohort of healthy individuals (Fig. 1f,g) we further focused on the outliers in the different age groups. Here, we used our previously defined threshold values for increased radiosensitivity [19, 28]. In the healthy individuals cohort there was a clear increase of outliers with age in the group of individuals having B/M values higher than 0.5 and individuals with B/M values higher than 0.6 (ρ = 0.9, *p* = 0.037). Each year the percentage of individuals with a B/M value higher than 0.6, grows by 0.64% (Fig. 2g). In the cancer patient cohort there was a high proportion of individuals having values higher than 0.5 B/M, yet without an increase with age. However the proportion of cancer patients with values higher than 0.6 B/M increased clearly with age, each year by 0.76% (*ρ* = 0.9, *p* = 0.037) (Fig. 2g).

**Fig. 3:**
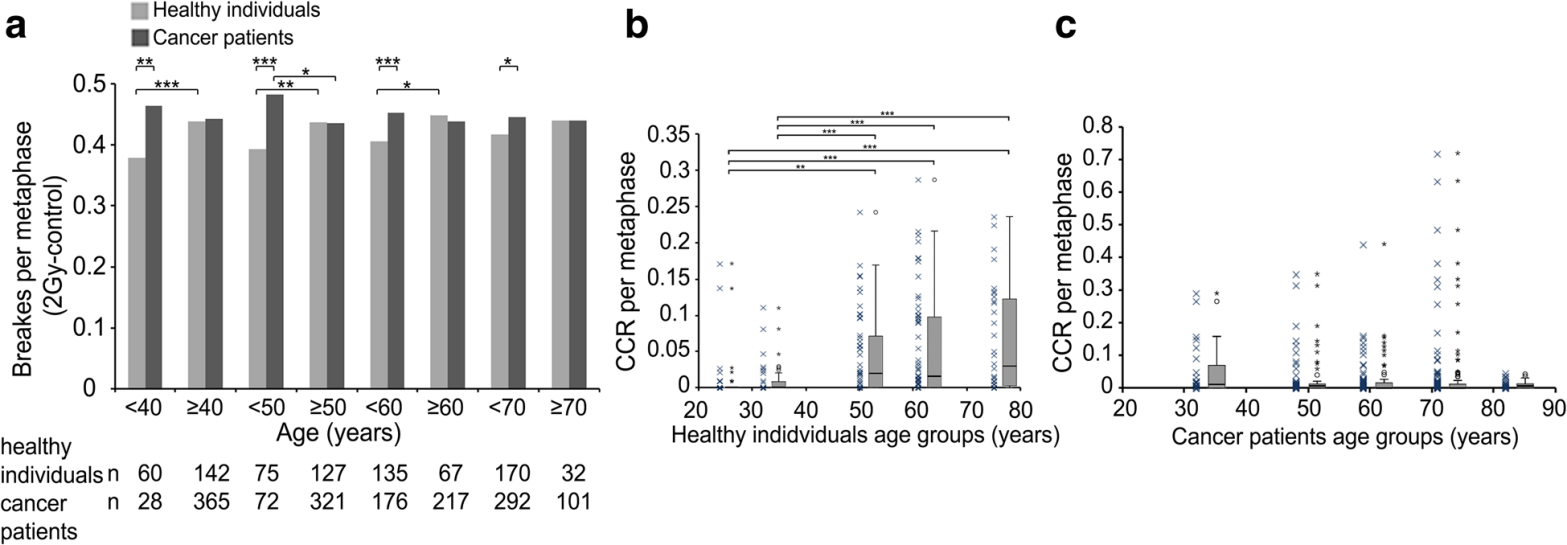
Radiosensitivity compared between healthy individuals and cancer patients and the occurrence of CCR dependent on age. Averaged radiosensitivity in healthy individuals younger than 40, 50, 60 or 70 years or equal or greater than 40, 50, 60, 70 years, compared to patients within the same age groups (**a**). Complex chromosome rearrangements per metaphase in healthy individuals (**b**) and cancer patients (**c**) after irradiation with a dose of 2 Gy. P-values ≤0.05 are marked with one asterisk, *P*-values ≤0.01 are marked with two

Next we were interested whether there is an age cut off for the B/M values between healthy individuals and patients. We categorized subgroups comprising younger and equal or older individuals from 40 to 70 years at 10 year intervals. Among the young groups healthy individuals had distinctly lower B/M values than cancer patients at age 40 (*p* = 0.007), 50 (*p* < 0.001), 60 (*p* = 0.001) and 70 (*p* = 0.013). But there was no difference between healthy individuals and patients of groups equal or older than 40, 50, 60 and 70 years (Fig. 3a). In the different age groups younger healthy individuals had lower B/M values than older and exactly the opposite is the case in younger cancer patients having higher B/M values than older. These differences decreased in the older age groups (Fig. 3a).

### Complex chromosomal aberrations and age

It was previously published that complex chromosomal aberrations are especially suited to predict individual radiosensitivity [28]. So we studied CCR in both groups separately. In healthy individuals CCR per metaphase (CCR/M) clearly increased between the age group 24 and 50 (*p* = 0.003), 24 and 61 (*p* < 0.001) and 24 and 75 years (*p* = 0.001) (Fig. 3b). Between age groups of cancer patients CCR/M there was no difference (Fig. 3c).

## Discussion

We used the 3C-FiSH to analyze chromosomal aberrations after ex vivo irradiation. A healthy individual cohort and a cancer patient cohort were studied. In the cancer patient cohort a considerable portion of individuals with increased radiosensitivity was expected, because individuals with impaired DNA damage processing are more prone to cancer [31]. About 20% of all cancers are assumed to have a genetic background [32] divided in inherited high penetrance genes and familial low penetrance genes with polygenetic mechanisms. In breast cancer up to 50% of cases might be related to a genetic cause [33]. Especially in young cancer patients due to their higher proportion of genetically caused cancers [34], a high amount of cancer patients with increased radiosensitivity was assumed. The consequence is that a relationship between age and radiosensitivity was not found.

So we used a second cohort of healthy individuals with no history of malignant disease. Here, a clear increase of the individual radiosensitivity with age was found. However, this mild overall increase will probably not increase radiotherapy related side effects. In the cancer patient cohort no such age dependent increase of radiosensitivity was found. The reason may be that at young age the proportion of genetically caused cancers is higher compared to older individuals. At older age there are probably still higher amounts of genetically induced cancers, however the portion of sporadic cancers without an increased radiosensitivity is higher than in the young age group.

More important for clinical radiotherapy and the occurrence of treatment related side effects may be the amount of outliers in the different age groups. We assume a distinctly increased radiosensitivity starting with a B/M of 0.6 and recommend reducing the radiation dose in these patients [26, 35, 36]. None of the young healthy individuals had a B/M value above 0.6 whereas 6.3% had increased B/M values at age 75. In the cancer patient cohort at age 31 already 10.3% of patients had B/M values above 0.6 and this portion rose to 20.8% at age 82. So the fraction of cancer patients with distinctly increased radiosensitivity clearly increased with age. There are only limited studies dealing with chromosomal radiosensitivity and age. A study comparing radiosensitivity of ten new borns using umbilical cord blood compared with twenty adults did not find an increase with age, but a distinct higher radiosensitivity of newborns [37]. Similar in a group of 14 individuals no influence of age on dicentric aberrations was found [38]. In a breast cancer cohort of 100 patients age at onset of disease did not correlate with radiosensitivity [39]. In a former study we found in a cohort of 67 breast cancer patients a high number of radiosensitive individuals between age 40 and 50 and afterwards a steep decline of radiosensitivity followed by a slow age dependent increase of radiosensitivity [26].

We used this 3-C FiSH approach because chromosomal aberrations in lymphocytes are particularly suited for the study of individual radiosensitivity. The advantage in analyzing aberrations is that all lymphocytes are irradiated in the G0 phase and therefore have no cell cycle dependent differences in radiosensitivity. After ex vivo irradiation lymphocytes have to process DNA damage and progress through the complete cell cycle up to mitosis. Additionally the lymphocytes may induce cell death. So all these lymphocytic abilities influence the chromosomal aberrations and therefore an impairment of these abilities will increase chromosomal aberrations and indicate increased radiosensitivity. Staining the chromosomes by fluorescent whole-chromosome (FISH) painting has the advantage that translocations and insertions are easily detected compared to conventional staining. We use a painting approach of chromosomes 1, 2 and 4 [35]. The three chromosomes are stained in red, green and yellow and are easily to distinguish and even small breaks or insertions of about 5 Mbp can be identified [40]. Additionally the three chromosomes represent 23% of the genomic DNA [41] and are involved in about 34% of all aberrations [42]. Painting all chromosomes by 24-C FiSH is challenging and the analyzing of the aberrations is even more complex [43]. Because by 3-C FiSH only a fraction of all possible exchanges are detected, the number of metaphases examined is adjusted accordingly and results in equivalent frequencies of chromosome exchanges [42].

## Conclusions

Individual radiosensitivity rises continuously with age, yet with strong interindividual variation. In cancer patients an age related increase of the individual radiosensitivity cannot be demonstrated due to the strong interindividual variation. However among old cancer patients there is a higher probability to have patients with clear increased radiosensitivity than in younger age.

## Additional file

Additional file 1: **Figure S1.** Three color fluorescence in situ hybridization of three chromosomes to determine radiosensitivity. Chromosome # 1 (red, rhodamine), chromosome # 2 (green, FITC) and chromosome # 4 (yellow, rhodamine + FITC) were painted with chromosome specific probes. DNA was stained with DAPI (blue) (A-D). The first row shows an overlay of the color images. In the second row are black and white images of the red painted chromosomes, in the third row of the green painted chromosomes. The last row displays all chromosomes of the blue stain as black and white images. All aberrations are marked in each color image where they occur. A normal metaphase spread is shown in (A). Metaphase spread with two translocations of # 2 with a blue chromosome and # 4 with a blue chromosome and an insertion of # 1 into a blue chromosome. The three aberrations were scored as 7 breaks (B). Metaphase spread with a dicentric chromosome of # 1 and # 2, a translocation of # 1 and # 4 and an insertion of # 1, # 2 and # 4. The aberrations were scored as 5 breaks (C). Metaphase spread with complex chromosomal rearrangements (CCR). The aberrations were scored as 12 breaks. The breaks of the red chromosome are marked with an arrow, of the green chromosome with an arrowhead and of the yellow one with a concave arrowhead (D). Scale: 10 μm. (PDF 947 kb)

## Abbreviations

3C-FiSH: 3 color-Fluorescence in situ hybridization
B/M: Breaks per metaphase
CCR: Complex chromosome rearrangement
CCR/M: Complex chromosome rearrangements per metaphase
DAPI: 4′,6-Diamidin-2-phenylindol
DNA: Deoxyribonucleic Acid
FiSH: Fluorescence in situ hybridization
FITC: Fluorescein isothiocyanate
Gy: Gray
h: hour
ICD: International classification of diseases
n: Sample number
