# Supplementary material for: Rate of individuals with clearly increased radiosensitivity rise with age both in healthy individuals and in cancer patients
Source: BMC Geriatr. 2018 May 4;18:105. doi: 10.1186/s12877-018-0799-y (PMC5935967; doi:10.1186/s12877-018-0799-y)
Supplement: Supplementary file 1 — Figure S1. Three color fluorescence in situ hybridization of three chromosomes to determine radiosensitivity. Chromosome # 1 (red, rhodamine), chromosome # 2 (green, FITC) and chromosome # 4 (yellow, rhodamine + FITC) were painted with chromosome specific probes. DNA was stained with DAPI (blue) (A-D). The first row shows an overlay of the color images. In the second row are black and white images of the red painted chromosomes, in the third row of the green painted chromosomes. The last row displays all chromosomes of the blue stain as black and white images. All aberrations are marked in each color image where they occur. A normal metaphase spread is shown in (A). Metaphase spread with two translocations of # 2 with a blue chromosome and # 4 with a blue chromosome and an insertion of # 1 into a blue chromosome. The three aberrations were scored as 7 breaks (B). Metaphase spread with a dicentric chromosome of # 1 and # 2, a translocation of # 1 and # 4 and an insertion of # 1, # 2 and # 4. The aberrations were scored as 5 breaks (C). Metaphase spread with complex chromosomal rearrangements (CCR). The aberrations were scored as 12 breaks. The breaks of the red chromosome are marked with an arrow, of the green chromosome with an arrowhead and of the yellow one with a concave arrowhead (D). Scale: 10 μm. (PDF 947 kb) [file 12877_2018_799_MOESM1_ESM.pdf]

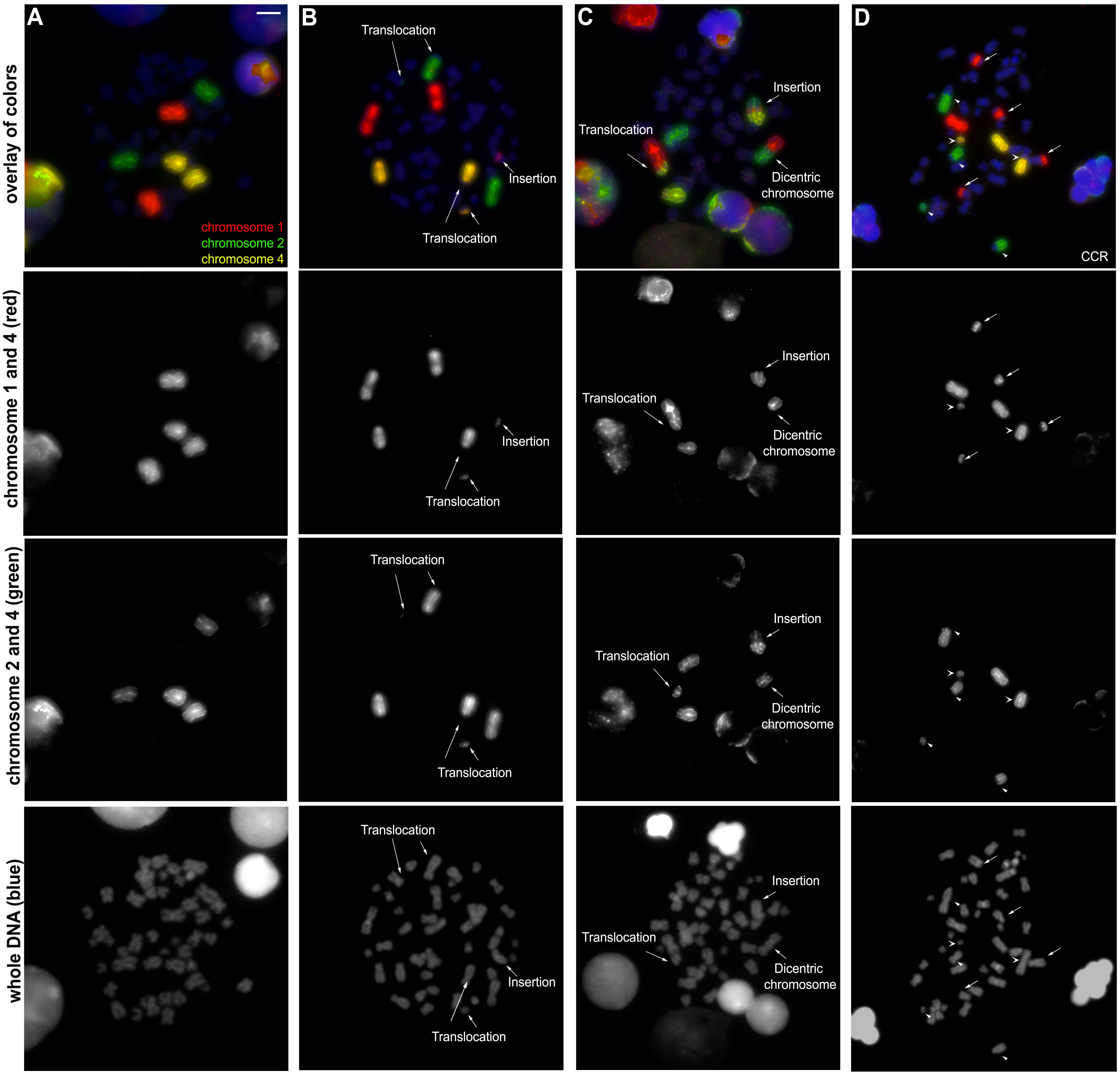
A
B
C
D
overlay of colors
chromosome 1 and 4 (red)
chromosome 2 and 4 (green)
whole DNA (blue)
chromosome 1
chromosome 2
chromosome 4
Translocation
Insertion
Dicentric chromosome
CCR

Additional figure 1: Three color fluorescence in situ hybridization of three chromosomes to determine radiosensitivity. Chromosome # 1 (red, rhodamine), chromosome # 2 (green, FITC) and chromosome # 4 (yellow, rhodamine + FITC) were painted with chromosome specific probes. DNA was stained with DAPI (blue) (A-D). The first row shows an overlay of the color images. In the second row are black and white images of the red painted chromosomes, in the third row of the green painted chromosomes. The last row displays all chromosomes of the blue stain as black and white images. All aberrations are marked in each color image where they occur. A normal metaphase spread is shown in (A). Metaphase spread with two translocations of # 2 with a blue chromosome and # 4 with a blue chromosome and an insertion of # 1 into a blue chromosome. The three aberrations were scored as 7 breaks (B). Metaphase spread with a dicentric chromosome of # 1 and # 2, a translocation of # 1 and # 4 and an insertion of # 1, # 2 and # 4. The aberrations were scored as 5 breaks. (C). Metaphase spread with complex chromosomal rearrangements (CCR). The aberrations were scored as 12 breaks. The breaks of the red chromosome are marked with an arrow, of the green chromosome with an arrowhead and of the yellow one with a concave arrowhead (D). Scale: 10µm.
